# COVID-19 Vaccination and Non–COVID-19 Mortality Risk — Seven Integrated Health Care Organizations, United States, December 14, 2020–July 31, 2021

**DOI:** 10.15585/mmwr.mm7043e2

**Published:** 2021-10-29

**Authors:** Stanley Xu, Runxin Huang, Lina S. Sy, Sungching C. Glenn, Denison S. Ryan, Kerresa Morrissette, David K. Shay, Gabriela Vazquez-Benitez, Jason M. Glanz, Nicola P. Klein, David McClure, Elizabeth G. Liles, Eric S. Weintraub, Hung-Fu Tseng, Lei Qian

**Affiliations:** ^1^Research and Evaluation, Kaiser Permanente Southern California, Pasadena, California; ^2^CDC COVID-19 Response Team; ^3^HealthPartners Institute, Minneapolis, Minnesota; ^4^Institute for Health Research, Kaiser Permanente Colorado, Denver, Colorado; ^5^Kaiser Permanente Vaccine Study Center, Kaiser Permanente Northern California, Oakland, California; ^6^Marshfield Clinic Research Institute, Marshfield, Wisconsin; ^7^Center for Health Research, Kaiser Permanente Northwest, Portland, Oregon; ^8^Immunization Safety Office, CDC.

By September 21, 2021, an estimated 182 million persons in the United States were fully vaccinated against COVID-19.* Clinical trials indicate that Pfizer-BioNTech (BNT162b2), Moderna (mRNA-1273), and Janssen (Johnson & Johnson; Ad.26.COV2.S) vaccines are effective and generally well tolerated (*1*–*3*). However, daily vaccination rates have declined approximately 78% since April 13, 2021^†^; vaccine safety concerns have contributed to vaccine hesitancy (*4*). A cohort study of 19,625 nursing home residents found that those who received an mRNA vaccine (Pfizer-BioNTech or Moderna) had lower all-cause mortality than did unvaccinated residents (*5*), but no studies comparing mortality rates within the general population of vaccinated and unvaccinated persons have been conducted. To assess mortality not associated with COVID-19 (non–COVID-19 mortality) after COVID-19 vaccination in a general population setting, a cohort study was conducted during December 2020–July 2021 among approximately 11 million persons enrolled in seven Vaccine Safety Datalink (VSD) sites.^§^ After standardizing mortality rates by age and sex, this study found that COVID-19 vaccine recipients had lower non–COVID-19 mortality than did unvaccinated persons. After adjusting for demographic characteristics and VSD site, this study found that adjusted relative risk (aRR) of non–COVID-19 mortality for the Pfizer-BioNTech vaccine was 0.41 (95% confidence interval [CI] = 0.38–0.44) after dose 1 and 0.34 (95% CI = 0.33–0.36) after dose 2. The aRRs of non–COVID-19 mortality for the Moderna vaccine were 0.34 (95% CI = 0.32–0.37) after dose 1 and 0.31 (95% CI = 0.30–0.33) after dose 2. The aRR after receipt of the Janssen vaccine was 0.54 (95% CI = 0.49–0.59). There is no increased risk for mortality among COVID-19 vaccine recipients. This finding reinforces the safety profile of currently approved COVID-19 vaccines in the United States.

VSD, a collaborative project between CDC’s Immunization Safety Office and nine health care organizations, collects electronic health data, including information on vaccines, for specific studies. In this cohort study of VSD members aged ≥12 years, vaccination status through May 31, 2021 was determined. Index dates were assigned to all persons on the basis of the distribution of vaccination dates among vaccinated persons.^¶^ Person-time for unvaccinated persons included unvaccinated person-time before COVID-19 vaccination among COVID-19 vaccinees, and unvaccinated person-time of persons who did not receive a COVID-19 vaccine by May 31, 2021. To ensure comparable health care–seeking behavior among persons who received a COVID-19 vaccine and those who did not (unvaccinated persons), eligible unvaccinated persons were selected from among those who received ≥1 dose of influenza vaccine in the last 2 years. Separate unvaccinated groups were selected for mRNA and Janssen vaccines.** Deaths were identified through VSD, which captures hospital deaths and deaths reported to health plans. In this study, non–COVID-19 deaths were assessed because a protective effect of COVID-19 vaccination for COVID-19–related deaths was expected. Non–COVID-19 deaths were those that did not occur within 30 days of an incident COVID-19 diagnosis or receipt of a positive test result for SARS-CoV-2 (the virus that causes COVID-19) via reverse transcription–polymerase chain reaction or rapid test.

Standardized mortality rates (SMRs) (deaths per 100 person-years) were calculated and compared with a rate ratio test between vaccinated and unvaccinated groups (*6*); a population of VSD members who were enrolled in December 2020 was used as the standard population. Overall SMRs were reported separately for Pfizer-BioNTech, Moderna, and Janssen vaccines. Poisson models were used to calculate overall aRRs and 95% CIs adjusted for age, sex, race and ethnicity, and VSD site. SMRs and aRRs by age, sex, and race and ethnicity were also calculated, adjusting for other demographic characteristics. Analytical units were aggregated counts of deaths and person-years by vaccination status, age, sex, race and ethnicity, and VSD site. All analyses were conducted using SAS statistical software (version 9.4; SAS Institute).^††^ This work was reviewed by CDC and VSD sites^§§^ and was conducted consistent with applicable federal law and CDC policy.^¶¶^

The cohort consisted of 6.4 million COVID-19 vaccinees and 4.6 million unvaccinated persons with similar characteristics as the comparison groups. Among 3.5 million Pfizer-BioNTech vaccine recipients, 9.2% were aged 12–17 years, 69.4% were aged 18–64 years, 54.0% were female, 42.7% were White persons, 21.4% were Hispanic persons, 16.6% were Asian persons, and 5.1% were Black persons (Table 1). Among 2.6 million Moderna vaccine recipients, 71.7% were aged 18–64 years, 54.5% were female, 44.2% were White persons, 23.1% were Hispanic persons, 14.2% were Asian persons, and 5.6% were Black persons. Among 342,169 Janssen vaccine recipients, 87.5% were aged 18–64 years, 4.1% were aged ≥75 years, 48.0% were female, 45.1% were White persons, 20.3% were Hispanic persons, 13.4% were Asian persons, and 6.1% were Black persons.

**TABLE 1:** Demographic characteristics of COVID-19 vaccine recipients and unvaccinated comparison group — seven integrated health care organizations, United States, December 14, 2020–July 31, 2021

Characteristic	No. (%)				
	mRNA vaccine*			Janssen vaccine	
	Pfizer-BioNTech vaccine recipients	Moderna vaccine recipients	Unvaccinated comparison group^†,§^	Janssen vaccine recipients	Unvaccinated comparison group^†^
**Total**	**3,452,126 (100.0)**	**2,604,066 (100.0)**	**3,243,112 (100.0)**	**342,169 (100.0)**	**1,346,445 (100.0)**
**Age group, yrs**					
12–17	316,587 (9.2)	NA	311,445 (9.6)	NA	NA
18–44	1,322,147 (38.3)	951,899 (36.6)	1,153,735 (35.6)	141,317 (41.3)	558,996 (41.5)
45–64	1,072,819 (31.1)	913,075 (35.1)	987,703 (30.5)	158,157 (46.2)	624,106 (46.4)
65–74	440,879 (12.8)	454,391 (17.4)	468,679 (14.5)	28,721 (8.4)	109,143 (8.1)
75–84	219,888 (6.4)	216,968 (8.3)	233,870 (7.2)	9,835 (2.9)	37,745 (2.8)
≥85	79,806 (2.3)	67,733 (2.6)	87,680 (2.7)	4,139 (1.2)	16,455 (1.2)
**Sex**					
Male	1,586,867 (46.0)	1,185,265 (45.5)	1,395,196 (43.0)	177,867 (52.0)	696,190 (51.7)
Female	1,865,259 (54.0)	1,418,801 (54.5)	1,847,916 (57.0)	164,302 (48.0)	650,255 (48.3)
**Race/Ethnicity**					
Hispanic	738,931 (21.4)	600,654 (23.1)	871,863 (26.9)	69,602 (20.3)	329,921 (24.5)
White, non-Hispanic	1,472,716 (42.7)	1,151,826 (44.2)	1,397,345 (43.1)	154,188 (45.1)	585,489 (43.5)
Asian, non-Hispanic	573,754 (16.6)	369,069 (14.2)	432,782 (13.3)	45,909 (13.4)	200,430 (14.9)
Black, non-Hispanic	175,066 (5.1)	145,127 (5.6)	189,592 (5.8)	20,996 (6.1)	73,174 (5.4)
Multiple races/Other/Unknown	491,659 (14.2)	337,390 (13.0)	351,530 (10.8)	51,474 (15.0)	157,431 (11.7)

**Abbreviations**: Janssen = Johnson & Johnson; NA = not applicable.

* Among Pfizer-BioNTech COVID-19 vaccine recipients, 2,980,152 received the second dose by May 31, 2021; among Moderna COVID-19 vaccine recipients, 2,362,157 received the second dose by May 31, 2021.

^†^ Unvaccinated comparison group included unvaccinated persons and COVID-19 vaccine recipients before COVID-19 vaccination. The assignment of index dates allowed COVID-19 vaccinees to contribute unvaccinated person-time before vaccination, thus avoiding immortal time bias.

^§^ mRNA vaccines included Pfizer-BioNTech and Moderna COVID-19 vaccines.

After excluding COVID-19–associated deaths, overall SMRs after dose 1 were 0.42 and 0.37 per 100 person-years for Pfizer-BioNTech and Moderna, respectively, and were 0.35 and 0.34, respectively, after dose 2 (Table 2). These rates were lower than the rate of 1.11 per 100 person-years among the unvaccinated mRNA vaccine comparison group (p <0.001). Among Janssen vaccine recipients, the overall SMR was 0.84 per 100 person-years, lower than the rate of 1.47 per 100 person-years among the unvaccinated comparison group (p <0.001). Among persons aged 12–17 years, SMRs were similar among the Pfizer-BioNTech vaccine recipients and unvaccinated comparison groups (p = 0.68 after dose 1 and 0.89 after dose 2). SMRs were also similar between Janssen vaccine recipients and unvaccinated comparison groups among Asian persons (p = 0.11). Among other subgroups defined by vaccine received, age, sex, and race and ethnicity, COVID-19 vaccine recipients had lower SMRs than did their unvaccinated counterparts (p <0.05).

**TABLE 2:** Number of deaths and standardized mortality rate (deaths per 100 person-years) not associated with COVID-19 among COVID-19 vaccine recipients and unvaccinated comparison groups, by age, sex, and race/ethnicity — seven integrated health care organizations, United States, December 14, 2020–July 31, 2021

Characteristic	No. of deaths* (standardized mortality rate per 100 person-years)						
	mRNA vaccine					Janssen vaccine	
	Pfizer-BioNTech vaccine recipients^†^		Moderna vaccine recipients^†^		Unvaccinated comparison group^§^	Vaccine recipients^¶^	Unvaccinated comparison group^§^
	After dose 1	After dose 2	After dose 1	After dose 2			
Overall**	**1,157 (0.42)**	**5,143 (0.35)**	**1,202 (0.37)**	**4,434 (0.34)**	**6,660 (1.11)**	**671 (0.84)**	**2,219 (1.47)**
**Age group,^††^ yrs**							
12–17	2 (0.01)	3 (0.01)	NA	NA	7 (0.01)	NA	NA
18–44	20 (0.02)	73 (0.02)	24 (0.03)	57 (0.02)	161 (0.07)	19 (0.04)	63 (0.08)
45–64	117 (0.16)	409 (0.13)	123 (0.16)	421 (0.17)	910 (0.51)	130 (0.25)	497 (0.66)
65–74	235 (0.79)	994 (0.62)	249 (0.63)	920 (0.58)	1,407 (2.13)	144 (1.49)	466 (2.77)
75–84	338 (2.32)	1,591 (1.89)	376 (2.00)	1,425 (1.77)	1,861 (6.34)	176 (5.59)	549 (9.13)
≥85	445 (7.90)	2,073 (6.85)	430 (7.16)	1,611 (6.57)	2,314 (18.76)	202 (15.35)	644 (23.76)
**Sex** ^§§^							
Male	587 (0.49)	2,584 (0.41)	640 (0.45)	2,352 (0.42)	3,265 (1.30)	326 (0.96)	1,102 (1.68)
Female	570 (0.35)	2,559 (0.29)	562 (0.30)	2,082 (0.28)	3,395 (0.96)	345 (0.75)	1,117 (1.31)
Race/Ethnicity**							
Hispanic	144 (0.36)	584 (0.29)	197 (0.35)	701 (0.33)	1,230 (1.07)	92 (0.91)	365 (1.24)
White, non-Hispanic	781 (0.47)	3,560 (0.39)	732 (0.39)	2,804 (0.37)	3,993 (1.17)	416 (0.85)	1,364 (1.58)
Asian, non-Hispanic	72 (0.23)	408 (0.23)	67 (0.18)	317 (0.21)	460 (0.78)	56 (0.83)	157 (1.09)
Black, non-Hispanic	84 (0.54)	300 (0.37)	130 (0.65)	340 (0.44)	623 (1.53)	65 (0.99)	187 (1.97)
Multiple races/Other/Unknown	76 (0.38)	291 (0.28)	76 (0.32)	272 (0.29)	354 (0.82)	42 (0.68)	146 (1.22)

**Abbreviations**: Janssen = Johnson & Johnson; NA = not applicable.

* Number of deaths as of July 31, 2021; deaths that occurred ≤30 days after an incident COVID-19 diagnosis or receipt of a positive SARS-CoV-2 test result were excluded.

^†^ Vaccinated with mRNA COVID-19 vaccines during December 14, 2020–May 31, 2021.

^§^ Unvaccinated comparison group included unvaccinated persons and COVID-19 vaccine recipients before COVID-19 vaccination. The assignment of index dates allowed COVID-19 vaccinees to contribute unvaccinated person-time before vaccination, thus avoiding immortal time bias.

^¶^ Vaccinated with Janssen COVID-19 vaccine during February 27, 2021–May 31, 2021.

** Overall mortality rates and race- and ethnicity-specific mortality rates were age- and sex-standardized.

^††^ Age-specific mortality rates were sex-standardized.

^§§^ Sex-specific mortality rates were age-standardized.

The overall aRR among Pfizer-BioNTech vaccine recipients compared with the unvaccinated comparison group was 0.41 (95% CI = 0.38–0.44) after dose 1 and 0.34 (95% CI = 0.33–0.36) after dose 2 (Table 3). Among Pfizer-BioNTech vaccine recipients aged 12–17 years, mortality risk among vaccinated and unvaccinated persons was similar after dose 1 (aRR = 0.85; 95% CI = 0.38–1.90) and after dose 2 (aRR = 0.73; 95% CI = 0.33–1.64). Among other age groups, aRRs ranged from 0.35 (95% CI = 0.29–0.42) among persons aged 45–64 years to 0.46 (95% CI = 0.39–0.54) among persons aged ≥85 years after dose 1, and from 0.28 (95% CI = 0.25–0.31) among persons aged 45–64 years to 0.39 (95% CI = 0.36–0.43) among those aged ≥85 years after dose 2. Similar aRRs among vaccinated persons compared with the unvaccinated comparison group were observed for recipients of the Moderna vaccine, ranging from 0.31 (95% CI = 0.26–0.37) among persons aged 45–64 years to 0.46 (95% CI = 0.31–0.69) among persons aged 18–44 years after dose 1, and 0.28 (95% CI = 0.26–0.32) among persons aged 65–74 years to 0.38 (95% CI = 0.29–0.50) among those aged 18–44 years after dose 2. The overall aRR for Janssen was 0.54 (95% CI = 0.49–0.59), and age-stratified aRRs ranged from 0.40 (95% CI = 0.34–0.49) among persons aged 45–64 years to 0.68 (95% CI = 0.56–0.82) among persons aged ≥85 years. Across vaccine type and dose, males and females had comparable aRRs. All vaccinated racial and ethnic groups had lower mortality risks than did unvaccinated comparison groups.

**TABLE 3:** Adjusted relative risks for mortality of COVID-19 vaccine recipients and unvaccinated comparison groups*— seven integrated health care organizations, United States, December 14, 2020–July 31, 2021

Characteristic	Vaccine type, aRR, (95% CI)				
	Pfizer-BioNTech		Moderna		Janssen
	After dose 1	After dose 2	After dose 1	After dose 2	After dose 1
**Overall^†^**	**0.41 (0.38–0.44)**	**0.34 (0.33–0.36)**	**0.34 (0.32–0.37)**	**0.31(0.30–0.33)**	**0.54 (0.49–0.59)**
**Age group,^§^ yrs**					
12–17	0.85 (0.38–1.90)	0.73 (0.33–1.64)	NA	NA	NA
18–44	0.37 (0.24–0.57)	0.36 (0.28–0.46)	0.46 (0.31–0.69)	0.38 (0.29–0.50)	0.55 (0.36–0.82)
45–64	0.35 (0.29–0.42)	0.28 (0.25–0.31)	0.31 (0.26–0.37)	0.33 (0.29–0.37)	0.40 (0.34–0.49)
65–74	0.39 (0.33–0.47)	0.32 (0.29–0.35)	0.32 (0.27–0.37)	0.28 (0.26–0.32)	0.50 (0.39–0.63)
75–84	0.38 (0.33–0.46)	0.32 (0.29–0.35)	0.32 (0.27–0.38)	0.29 (0.26–0.32)	0.58 (0.48–0.71)
≥85	0.46 (0.39–0.54)	0.39 (0.36–0.43)	0.38 (0.32–0.45)	0.35 (0.31–0.39)	0.68 (0.56–0.82)
**Sex** ^¶^					
Male	0.41 (0.37–0.46)	0.35 (0.33–0.38)	0.36 (0.32–0.40)	0.33 (0.31–0.35)	0.52 (0.46–0.60)
Female	0.41 (0.36–0.45)	0.33 (0.31–0.36)	0.33 (0.29–0.37)	0.30 (0.28–0.32)	0.56 (0.49–0.64)
Race/Ethnicity**					
Hispanic	0.36 (0.30–0.42)	0.29 (0.26–0.32)	0.33 (0.29–0.39)	0.31 (0.28–0.34)	0.58 (0.46–0.73)
White, non-Hispanic	0.44 (0.38–0.50)	0.37 (0.34–0.40)	0.35 (0.30–0.40)	0.32 (0.30–0.35)	0.53 (0.46–0.61)
Asian, non-Hispanic	0.31 (0.25–0.39)	0.32 (0.28–0.36)	0.23 (0.18–0.30)	0.27 (0.23–0.30)	0.68 (0.52–0.88)
Black, non-Hispanic	0.38 (0.31–0.47)	0.27 (0.24–0.31)	0.42 (0.35–0.49)	0.29 (0.25–0.32)	0.47 (0.36–0.63)
Multiple races/Other/Unknown	0.46 (0.36–0.60)	0.35 (0.30–0.41)	0.40 (0.30–0.51)	0.36 (0.30–0.42)	0.52 (0.38–0.71)

**Abbreviations**: aRR = adjusted relative risk; CI = confidence interval; Janssen = Johnson & Johnson; NA = not applicable; VSD = Vaccine Safety Datalink.

* Unvaccinated comparison groups included unvaccinated persons and COVID-19 vaccine recipients before COVID-19 vaccination. The assignment of index dates allowed COVID-19 vaccinees to contribute unvaccinated person-time before vaccination, thus avoiding immortal time bias.

^†^ Overall relative risks were adjusted for age, sex, race and ethnicity, and VSD site.

^§^ Relative risks by age were adjusted for sex, race and ethnicity, and VSD site.

^¶^ Relative risks by sex were adjusted for age, race and ethnicity, and VSD site.

** Relative risks by race and ethnicity were adjusted for age, sex, and VSD site.

## Discussion

In a cohort of 6.4 million COVID-19 vaccinees and 4.6 million demographically similar unvaccinated persons, recipients of the Pfizer-BioNTech, Moderna, or Janssen vaccines had lower non–COVID-19 mortality risk than did the unvaccinated comparison groups. There is no increased risk for mortality among COVID-19 vaccine recipients. This finding reinforces the safety profile of currently approved COVID-19 vaccines in the United States. The lower mortality risk after COVID-19 vaccination suggests substantial healthy vaccinee effects (i.e., vaccinated persons tend to be healthier than unvaccinated persons) (*7*,*8*), which will be explored in future analyses. Mortality rates among Janssen vaccine recipients were not as low as those among mRNA vaccine recipients. This finding might be because of differences in risk factors, such as underlying health status and risk behaviors among recipients of mRNA and Janssen vaccines that might also be associated with mortality risk.

Among persons aged 12–17 years, mortality risk did not differ between Pfizer-BioNTech vaccinees and unvaccinated persons; only 12 deaths occurred in this age group during the study period. The unvaccinated group might be more similar to the vaccinated group in risk factors than are vaccinated and unvaccinated adults. Stratified analyses by age, sex, and race and ethnicity showed that vaccinated adults had lower mortality than did unvaccinated adults across subgroups.

The findings in this report are subject to at least four limitations. First, the study was observational, and individual-level confounders that were not adjusted for might affect mortality risk, including baseline health status, underlying conditions, health care utilization, and socioeconomic status. Second, healthy vaccinee effects were found in all but the youngest age group. Such effects were also found in a cohort study conducted in a nursing home population, which reported substantially lower aRRs for 7-day mortality among vaccinated residents after dose 1 (0.34) and dose 2 (0.49) as compared with unvaccinated residents (*5*). Lower rates of non–COVID-19 mortality in vaccinated groups suggest that COVID-19 vaccinees are inherently healthier or engage in fewer risk behaviors (*7*,*8*); future analyses will address these issues. Third, although deaths associated with COVID-19 were excluded, causes of death were not assessed. It is possible that the algorithm used might have misclassified some deaths associated with COVID-19 because of lack of testing or because individual mortality reviews were not conducted. Finally, the findings might not be applicable to the general population. The VSD includes approximately 3% of the U.S. population, and is representative of the general population with regard to several demographic and socioeconomic characteristics (*9*). Other studies have already demonstrated the safety of COVID-19 vaccines authorized in the United States. 

Despite these limitations, this study had several strengths. First, this was a cohort study with a large, sociodemographically diverse population, and it encompassed a study period of >7 months. Second, VSD sites were able to capture COVID-19 vaccines administered not just within but also outside their health care systems, including COVID-19 vaccine doses recorded in state immunization registries, allowing for more complete ascertainment of vaccination status. Third, the assignment of index dates allowed COVID-19 vaccinees to contribute unvaccinated person-time before vaccination, thus avoiding immortal time bias (*10*), which can confer a spurious survival advantage to the treatment group in cohort studies. Index date assignments made the follow-up period comparable between COVID-19 vaccinees and their comparators and helped control for seasonality and general trends in mortality.

CDC recommends that everyone aged ≥12 years should receive a COVID-19 vaccine to help protect against COVID-19.*** This cohort study found lower rates of non–COVID-19 mortality among vaccinated persons compared with unvaccinated persons in a large, sociodemographically diverse population during December 2020–July 2021. There is no increased risk for mortality among COVID-19 vaccine recipients. This finding reinforces the safety profile of currently approved COVID-19 vaccines in the United States.

**Summary**

**What is already known about this topic?**

Although deaths after COVID-19 vaccination have been reported to the Vaccine Adverse Events Reporting System, few studies have been conducted to evaluate mortality not associated with COVID-19 among vaccinated and unvaccinated groups.

**What is added by this report?**

During December 2020–July 2021, COVID-19 vaccine recipients had lower rates of non–COVID-19 mortality than did unvaccinated persons after adjusting for age, sex, race and ethnicity, and study site.

**What are the implications for public health practice?**

There is no increased risk for mortality among COVID-19 vaccine recipients. This finding reinforces the safety profile of currently approved COVID-19 vaccines in the United States. All persons aged ≥12 years should receive a COVID-19 vaccine.

## Footnotes

*
https://covid.cdc.gov/covid-data-tracker/#vaccinations

† https://ourworldindata.org/coronavirus (Accessed September 21, 2021).

§ Among nine VSD sites, (all health care organizations), data is included from seven sites: Kaiser Permanente (KP) Southern California, Pasadena, California; KP Northern California, Oakland, California; KP Colorado, Denver, Colorado; KP Northwest, Portland, Oregon; KP Washington, Seattle, Washington; HealthPartners, Minneapolis, Minnesota; and Marshfield Clinic, Marshfield, Wisconsin. Harvard Pilgrim Health Care Institute, Boston, Massachusetts, did not participate in this study because it is not a data-contributing site; Denver Health, Denver, Colorado, did not participate in this study because of limited resources.

¶ Persons who were vaccinated during December 14, 2020–May 31, 2021 were included in the vaccinated group. In each VSD site, age group, and sex stratum, the distribution of vaccination dates of dose 1 were obtained and used to assign index dates to all persons. Among vaccinated persons, if the index date was before the vaccination date of dose 1, follow-up started on the index date, and persons in this group contributed both unvaccinated person-time (from index date to the day before vaccination date) and vaccinated person-time (from vaccination date); if the index date was on or after the vaccination date of dose 1, follow-up started on the vaccination date, and persons in this group only contributed person-time after vaccination. Follow-up ended upon death, disenrollment from health plans, receipt of a COVID-19 vaccine for unvaccinated persons during June 1, 2021–July 31, 2021, or end of follow-up (July 31, 2021), whichever occurred first.

** All available eligible comparators were used for analysis of mRNA COVID-19 vaccines. Because the Janssen COVID-19 vaccine was authorized months after the mRNA COVID-19 vaccines and demographic characteristics of Janssen versus mRNA COVID-19 vaccine recipients might differ, a separate group of comparators was selected for Janssen vaccine recipients on the basis of calendar time and demographic characteristics of Janssen vaccine recipients. Because the number of Janssen vaccine recipients was smaller, four eligible comparators were randomly selected for each vaccinated individual to achieve optimal statistical power.

†† The procedure STDRATE was used to conduct rate ratio tests, and the procedure GENMOD was used to fit Poisson models.

§§ All activities were approved by the institutional review boards at some participating institutions or as public health surveillance activities at other participating institutions.

¶¶ 45 C.F.R. part 46.102(l)(2), 21 C.F.R. part 56; 42 U.S.C. Sect. 241(d); 5 U.S.C. Sect. 552a; 44 U.S.C. Sect. 3501 et seq.

*** https://www.cdc.gov/coronavirus/2019-ncov/vaccines/recommendations/adolescents.html (Accessed October 13, 2021).
